# Leukocyte Telomere Length as Potential Biomarker of HD Progression: A Follow-Up Study

**DOI:** 10.3390/ijms232113449

**Published:** 2022-11-03

**Authors:** Daniela Scarabino, Liana Veneziano, Elide Mantuano, Ivan Arisi, Alessia Fiore, Marina Frontali, Rosa Maria Corbo

**Affiliations:** 1 Institute of Molecular Biology and Pathology, National Research Council, 00185 Rome, Italy; 2 Institute of Translational Pharmacology, National Research Council, 00133 Rome, Italy; 3 Fondazione European Brain Research Institute (EBRI) Rita Levi-Montalcini, 00161 Rome, Italy; 4 Department of Biology and Biotechnology, La Sapienza University, 00185 Rome, Italy

**Keywords:** Huntington’s disease, leukocyte telomere length, neurodegenerative diseases, fluid biomarkers

## Abstract

The identification of biomarkers for neurodegenerative disorders such as Huntington’s disease (HD) is crucial for monitoring disease progression and therapeutic trial outcomes, especially in the pre-manifest disease stage (pre-HD). In a previous study, we observed that leukocyte telomere length (LTL) was strongly correlated with the estimated time to clinical onset in pre-HD subjects. To validate this hypothesis, we designed a follow-up study in which we analyzed LTL in 45 pre-HD stage subjects at baseline (T0) and then again after clinical onset at follow-up (T1); the follow-up interval was about 3 years, and the CAG range was 39–51 repeats; 90 peripheral blood mononuclear cell samples (PBMCs) were obtained from the Enroll-HD biorepository. In pre-HD subjects at T0, LTL was significantly reduced by 22% compared to the controls and by 14% from T0 at T1. No relationship was observed between the LTL and CAG numbers in subjects carrying different CAG repeats at T0 and at T1, suggesting that LTL reduction occurs independently of CAG number in pre-HD subjects. ROC curve analysis was used to test the validity of LTL as a potential biomarker of HD progression and showed that LTL measurement is extremely accurate in discriminating pre-HD subjects from the controls and even pre-HD from manifest HD, thus yielding a robust prognostic value in pre-HD subjects.

## 1. Introduction

Huntington’s disease (HD) is an autosomal dominant, progressive neurodegenerative disorder caused by a CAG trinucleotide repeat expansion in the first exon of the HTT gene encoding huntingtin (HTT), a ubiquitously expressed protein involved in transcriptional regulation [1,2]. The mutation leads to a pathological expanded polyglutamine stretch (polyQ) in the huntingtin, whose length varies depending on the CAG expansion number, which is toxic to the central nervous system.

The classic signs of HD are chorea, cognitive decline, and behavioral and psychiatric disturbances [3]. HD is fully penetrant in individuals with ≥ 40 repeats, with the onset of motor symptoms in middle age, often in the fourth or the fifth decade. Age at onset (AO) is negatively correlated with CAG repeat size, which accounts for approximately 56% of the variation in AO in the 40 to 55 CAG repeat range, typically associated with adult onset. The remaining variation in AO is due to genetic, stochastic, and environmental factors [4,5,6,7]. 

In individuals carrying the mutation, symptoms gradually emerge during a pre-manifest phase (pre-HD), but the timing of the transition from pre-manifest to manifest status is difficult to establish. Predicting disease progression in the pre-HD stage would be a relevant achievement as it could facilitate better planning of possible therapeutical interventions. Statistical models have been devised to estimate the age at disease onset from the length of an individual’s CAG repeat, but their accuracy is limited because the number of CAGs accounts for only a part of the variation in the onset of the disease [8,9]. Other biomarkers (biological, clinical, instrumental) have been studied to determine whether they can predict clinical onset of HD and track its progression [10,11]. Few validated biomarkers are currently available, however.

Human telomeres consist of tandem repeated TTAGGG nucleotide sequences located at the ends of chromosomes where they act as natural protective caps against DNA damage. During physiological DNA replication, telomeres progressively shorten with each cell division, due to the inability of the DNA polymerase to replicate the 3′ end of the DNA strand. Telomerase, a cellular ribonucleoprotein enzyme complex, counteracts telomere shortening. Though usually present in the early stages of embryonic development, its activity is almost absent in adult tissues, including skin, kidney, liver, blood vessels, and peripheral leukocytes [12,13]. As a consequence, the telomeres shorten progressively with advancing age in the replicating cells of adult tissues [14]. This phenomenon may indicate cellular senescence and an organism’s biological age. While telomere length varies across different tissue types, data from human studies have found strong correlations in telomere length across somatic tissues [15]. 

Peripheral blood mononuclear cells (PBMCs) are ideal for telomere research; they are easy to obtain from blood and are readily available. Furthermore, because they circulate throughout the body, immune cells are exposed to both internal (from cell) and external (from diet and exposures) factors affecting telomere maintenance. They are also involved in the interaction between psychological processes and the nervous and immune systems [16]. Shortened leukocyte telomere length (LTL) has been found to be associated with various diseases, including cardiovascular diseases [17], diabetes and metabolic syndrome [18,19], psychological disorders [20], and autoimmune diseases [21]. Reduced LTL can also be observed in neurodegenerative diseases such as Alzheimer’s disease (AD) [22,23,24,25], HD [26], and some forms of spinocerebellar ataxias (SCA) [27], whereas no consistent evidence of shorter telomere length in Parkinson disease (PD) has been found [28]. 

LTL in HD has been investigated in several studies and found to be shorter in HD patients than in controls [26,29,30,31]. In a previous study [26], we observed shorter leukocyte telomeres even in the pre-manifest HD (pre-HD) subjects compared to the controls. An analysis of the relationship between LTL in pre-HD and the estimated time to clinical diagnosis calculated according to the formula of Langbehn et al. [8] suggested that LTL in pre-manifest HD subjects could be a measure of time to clinical onset. The data also suggested that CAG number contributes to leukocyte telomere attrition in pre-HD. Since this hypothesis requires accurate validation through testing the relationship between LTL in the pre-manifest stage, CAG size, and actual years to clinical disease onset, we designed a follow-up study in which we analyzed LTL in HD subjects in the pre-manifest stage and then again after clinical onset in a CAG range from 39 to 51 repeats.

## 2. Results

Table 1 presents the sample characteristics of the controls and of the 45 HD patients examined at baseline (T0) in the pre-manifest stage and at follow-up (T1) in the manifest stage. The mean age of the pre-HD patients at baseline was 41.9 ± 10.4 years, the mean age of HD patients at follow-up was 45.5 ± 10.5 years, and the average follow-up interval was 3.4 ± 0.75 years (median 3, range 2–5). The mean age at clinical HD onset was 44.4 ± 10.7 years, indicating that the baseline pre-HD samples were collected about 2.5 (2.5 ± 0.99) years before HD clinical onset and that follow-up samples were collected about 0.9 (0.96 ± 1.2) years after onset.

**Figure 1:**
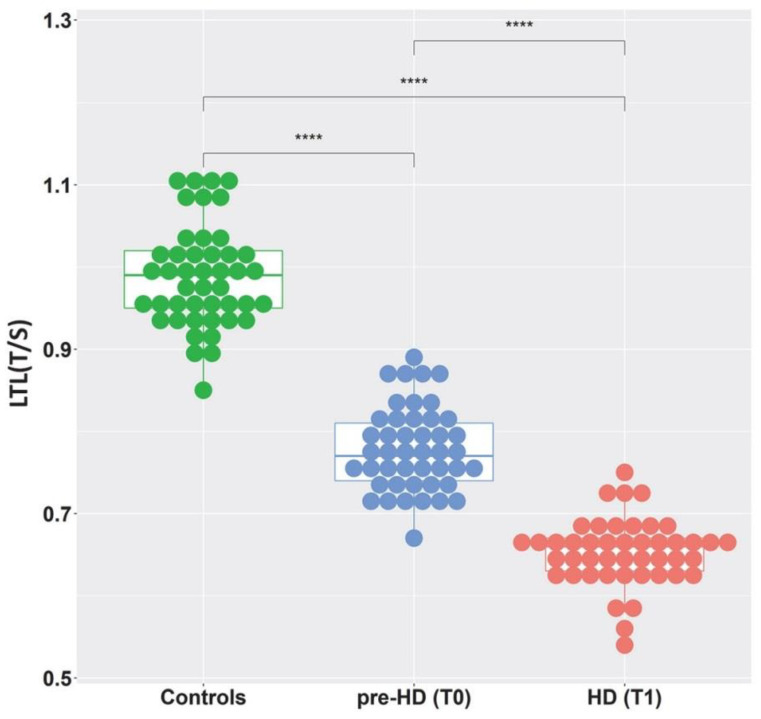
Distribution of LTL in controls, pre-HD, and HD patients. Box plot showing the distribution of LTL expressed as a relative telomere length T/S ratio (the number of copies of telomeric repeats T compared to a single-copy gene S) used as a quantitative control. LTL measures are presented for control subjects (green) and for patients in the pre-HD stage (blue) and the following corresponding HD stage (red). (**** = *p* < 0.0001).

LTL expressed as a T/S ratio was measured in 45 controls (median 0.99, Q1 0.95, Q3 1.02) and in patients at T0 (pre-HD stage) (median 0.77, Q1 0.74, Q3 0.81) and at T1 (manifest HD) (median 0.66, Q1 0.63, Q3 0.67) (Figure 1). The differences between the controls and the HD patients at baseline (pre-HD) were statistically significant (*p* < 0.0001), as were the differences between the HD patients at baseline and at follow-up, i.e., between the pre-manifest and manifest HD stages (*p* < 0.0001). A reduction in LTL of 0.13 ± 0.05 T/S, or about 0.04 T/S per year, between T0 and T1 was observed. 

**Figure 2:**
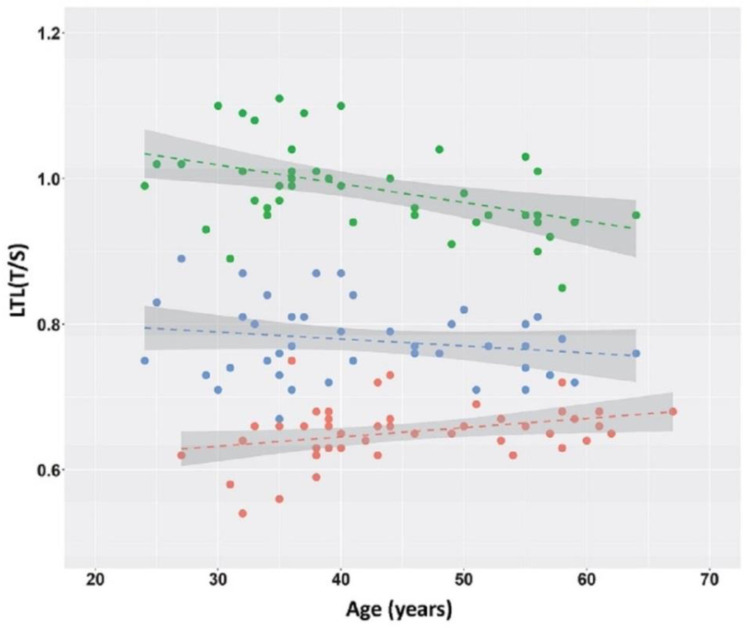
LTL values as a function of age at blood sampling. LTL measures expressed as a relative telomere length T/S ratio are presented for control subjects (green) and for patients in the pre-HD stage (blue) and the following corresponding HD stage (red). Linear regression lines are shown.

As LTL usually decreases with age in normal subjects, we analyzed the LTL/age relationship in the three samples (Figure 2). As expected, a negative relationship was observed between LTL and age in the controls (y = −0.0026 x + 1.1; *p* = 0.003; 95% confidence interval [CI] −0.00409 ≤ β ≤ −0.00111). No relationship was observed between LTL and age at baseline (pre-manifest HD) (*p* = 0.20), while there was a positive relationship between LTL and age at follow-up (manifest HD) where, however, the 95% CI of the regression coefficient was quite large (y = 0.001 x + 0.59; *p* = 0.03, 95% CI 0.00015 ≤ β ≤ 0.00241). A statistically significant relationship was observed between the follow-up interval and the LTL reduction (y = 0.04 x − 0.08; *p* = 0.0001, 95% CI 0.02160 ≤ β ≤ 0.05798), indicating that the longer the follow-up, the greater the reduction in LTL. 

**Figure 3:**
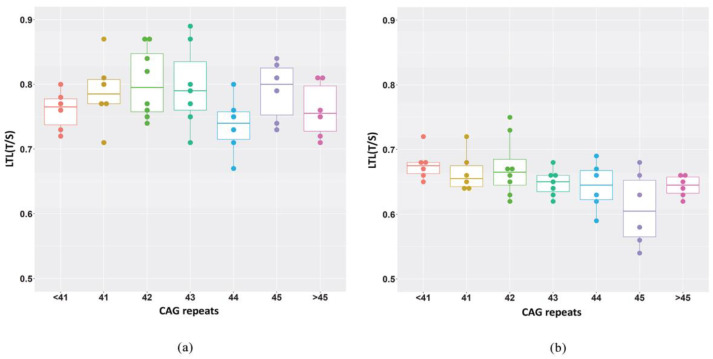
LTL values as a function of CAG number in patients. Box plots showing the distribution of LTL expressed as a relative telomere length T/S ratio: (**a**) at baseline (pre-HD stage) (*p* = 0.26); (**b**) at follow-up (manifest HD stage) (*p* = 0.15). Groups are compared with Kruskal–Wallis test.

We also wanted to determine the differences in LTL variation between HD patients carrying a different number of repeats. No difference in the median LTL of pre-manifest HD patients carrying different CAG repeat numbers was noted at about 2.5 years before disease clinical onset, even if the CAG repeat number was associated with a different age (Tab 2, Figure 3A), due to the negative relationship between CAG repeat size and age at onset. Similarly, no difference was noted between the LTL of manifest HD patients at about 1 year after clinical disease onset (Table 2, Figure 3B). A statistically significant difference in reduction in LTL was observed between HD patients carrying a different number of repeats after an average follow-up of 3.4 years. No clear trend was apparent. The post-hoc comparison test showed a statistically significant difference only between the 40 CAG group and the 45 CAG group (*p* < 0.05). 

**Figure 4:**
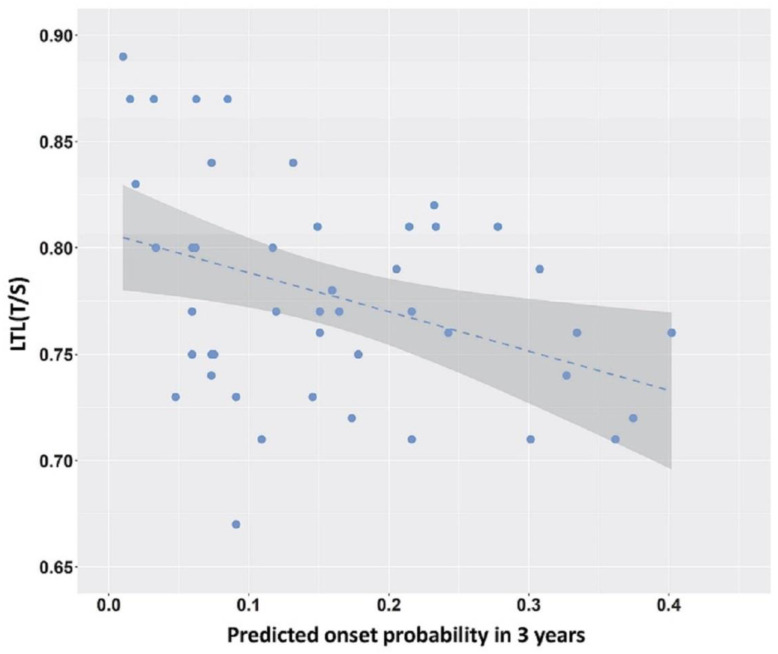
LTL values for pre-HD patients as a function of probability of disease onset in 3 years. The probability of onset was calculated according to Langbehn, 2004 [8]. Linear regression line is shown. Spearman correlation index r = −0.35, *p* = 0.016.

A correlation analysis between LTL at T0 and at T1, and clinical symptoms assessed using UHDRS-TMS and UHDRS-TFC scores at T0 and T1 revealed no statistically significant correlations (Table S1 in Supplementary Material). A statistically significant correlation was found between LTL in pre-HD patients and the probability of disease onset within 3 years (mean follow-up interval) calculated according to Langbehn, 2004 [8]. A reduction in LTL in pre-HD patients was significantly correlated with an increasing probability of clinical HD onset (r = −0.35, *p* = 0.016) (Figure 4).

**Figure 5:**
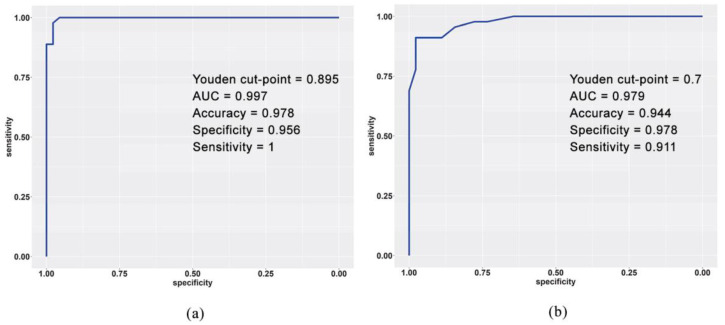
Assessment of LTL as a biomarker of HD. ROC curves show the performance to discriminate diagnostic groups based on LTL value: (**a**) pre-HD (T0) vs. controls; (**b**) pre-HD (T0) vs. manifest HD (T1). The AUC, best sensitivity, best specificity, and optimal cut-point based on Youden criterion are shown.

We then applied ROC curve analysis to test the accuracy of LTL as a biomarker. ROC analysis showed that an LTL cut-point of 0.895 (the Youden index criterion) had a sensitivity of 1.000 and a specificity of 0.956 (AUC 0.997) for distinguishing the pre-HD patients from the controls (Figure 5A). Comparison between the T0 (pre-HD) and T1 (manifest HD) cohorts yielded an LTL cut-point of 0.700 (AUC 0.979) with a sensitivity of 0.911 and a specificity of 0.978 for distinguishing pre-HD patients at about 3 years from clinical HD onset from manifest HD in the initial disease stage (Figure 5B).

## 3. Discussion

Our previous cross-sectional study suggested LTL as a good biomarker of HD conversion from the pre-manifest stage to overt disease [26]. To confirm this hypothesis, we performed a follow-up study with a temporal interval between two blood samplings of about 3 years, including the transition period from the pre-manifest stage to clinical onset and the initial manifest disease stage. At baseline (T0), in the pre-HD stage, approximately 2.5 years before clinical onset, LTL was reduced by 22% compared to the controls (*p* < 0.0001). At follow-up (T1), approximately 1 year after the clinical onset of HD, LTL values were further reduced by 14% from the baseline and 34% from the control LTL values. LTL in HD mutation carriers was examined in several studies [26,29,30,31], all showing significantly lower LTL in manifest HD subjects than observed in the controls. Our previous study [26] reported the first indication of reduced LTL in pre-HD patients. In the present study, we confirmed this result and found that the LTL in pre-HD patients has intermediate values between the controls and the manifest HD patients, being far shorter than in the controls and significantly longer than in the manifest HD patients over a follow-up interval of just 3 years. The LTL in the manifest HD patients (median 0.66 T/S) was markedly shortened compared to the other neurodegenerative diseases we studied to date: Alzheimer’s disease (mean LTL 0.70 T/S) [24], spinocerebellar ataxia 1 (SCA1) (median LTL 0.75 T/S), SCA2 (median LTL 1.06 T/S), and SCA3 (median LTL 0.90 T/S) [27]. There is ample evidence that leukocyte telomere shortening is a common hallmark of conditions associated with increased systemic oxidative stress and chronic inflammation [14]. The average LTL we observed in neurodegenerative diseases (SCA2 > SCA3 > AD > SCA1 > HD) could reflect an increasing relevance of chronic inflammation and/or systemic oxidative stress in their pathogenic processes. This hypothesis is in line with the observations that the expression of mHTT in the pathogenesis of HD results in neuroinflammation accompanied by chronic low-grade inflammation, which may provoke or exacerbate neurodegeneration, as well as produce systemic symptoms [32,33]. 

We also examined the relationship between LTL and age and CAG size. The CAG repeat range was 39 to 51 in the HD patients, and, as expected, the higher the number of CAG repeats, the lower the patient’s age (Table 2). No difference in LTL was observed at either T0 or T1 between HD patients carrying different CAG size alleles (Table 2, Figure 3*).* Similarly, no relationship was observed between LTL and age in pre-HD stage patients at T0, while a slight positive trend was found in manifest-HD-stage patients at follow-up (Figure 2). Differently, LTL analysis showed a clear negative relationship, as expected, between LTL and age due to the progressive telomere shortening with aging in the controls [14]. On the whole, our data indicate that at 3 years before clinical onset, the LTL is reduced in the pre-HD patients compared to the controls, and that LTL is independent of CAG number and age (Table 2). 

Our previous cross-sectional study involving 38 pre-HD patients (age range 19–62 years, CAG repeat range 40–52, unknown but presumably variable time to onset) indicated that telomere length depended on age and CAG number [26]. LTLs among pre-HD patients can be seen as the peripheral expression of mHTT-induced neuroinflammation, associated with neurological damage and microglia activation. Combining previous and present data, it could be hypothesized that in pre-HD subjects, LTL will depend on the number of CAGs when the clinical onset is still several years away, as it reflects the different degree of neurological damage caused by a different amount of CAG repeats. In contrast, whatever the duration of the pre-manifest stage, in the years just preceding the clinical onset (about 2–3 years), neurological damage is presumably similar in all HD subjects, and therefore the LTL will also be similar whatever the number of CAGs, converging towards the reduction typical of the disease. This picture may reflect what was observed in large clinical studies that reported an acceleration in disease progression just before symptom onset [34,35]. Nonetheless, this general model of LTL shortening in HD warrants confirmation both by increasing the sample size and within longitudinal studies, e.g., at 5 and 10 years before onset, to ascertain the real timing of telomere shortening. 

The LTL at follow-up, on average at about 1 year after the disease onset, was 66% of that of the controls, with a reduction of 0.13 T/S compared to the pre-HD stage, i.e., a telomere loss rate of about 0.04 T/S per year of follow-up. This rate is higher than the estimated rate of 0.01 T/S/years reported in healthy adults [36]. Previously, in a sample of HD patients with a median disease duration of 4 years (Q1 1.5, Q3 17), we observed that the LTL was 61% of that of the controls, and no major reduction in LTL was associated with disease duration [26]. This indicates that after greater attrition in the pre-manifest stage, LTL does not seem to undergo major shortenings in the years following the HD clinical onset, perhaps because it approaches the minimum length reported to be necessary to ensure human telomere protective stability in the PBMCs [14].

We noted no correlation between LTLs in the pre-HD or the HD stages and the clinical measures (TMS and TFC) (Table S1); however, we found an inverse correlation between a reduction in LTL and the probability of disease onset within 3 years (Figure 4). This would mean that LTL, rather than a marker of symptom presentation, may be a marker of pathogenic changes occurring in the pre-HD brain over a time interval proximal to the clinical onset. 

The role of LTL as a potential biomarker of HD progression, as validated with ROC analysis, showed a high accuracy of LTL in discriminating the pre-HD patients from the controls (AUC = 0.997), and even in discriminating pre-HD from manifest HD (AUC = 0.979). In the latter case, setting the cut-point of LTL at 0.700 T/S enables manifest HD to be correctly predicted at the initial stages with a sensitivity of 0.911 and a specificity of 0.978. We also compared the diagnostic performance of LTL with mHTT and neurofilaments (NfLs) from cerebral spinal fluid (CSF) and NfLs from peripheral blood, as described in Byrne et al., 2018 [37]. LTL seemed to outperform CSF mHTT (AUC 0.778), and plasmatic or intrathecal NfLs (AUC 0.931 and AUC 0.914, respectively) as a biomarker for pre-HD vs. manifest HD (AUC 0.979). 

## 4. Materials and Methods

### 4.1. Sample

The PBMCs of 45 HD patients, non-renewable material, were obtained from the ENROLL (https://www.enroll-hd.org/) (MTA (Material Transfer Agreement) Enroll-HD Biosamples Use Agreement Version RevNo002 (101216)). Two samples for each patient were collected during two visits about 3 years apart, for a total of 90 PBMC samples. The first sample at baseline (T0) was collected in an HD subject clinically classified at the pre-manifest stage in the Enroll database (pre-HD), and then the second sample at follow-up (T1) when the same patient was classified as manifest HD. The actual mean follow-up interval between T0 and T1 collection was 3.4 ± 0.7 years. The repeat range of mutated alleles varied from 39 to 51; there were about six patients per CAG repeat number (≤40, 41, 42, 43, 44, 45, ≥46), as the aim was to determine the differences in LTL variation between HD patients carrying a different number of repeats. Scores assessing motor function (Unified Huntington’s Disease Rating Scale—Total Motor Score, UHDRS-TMS) and functional capacity (UHDRS—Total Functional Capacity, TFC) were obtained from the Enroll database. Age at clinical onset was defined as the age at which progression from the pre-manifest to the manifest stage was clinically established, as reported in the Enroll database. The control samples, matched for age at baseline and sex, were healthy blood donors recruited at the Department of Experimental Medicine and Surgery at the University of Tor Vergata, Rome, Italy. The use of the control subjects sample was approved by the CNR Research Ethics and Integrity Committee (Ethical Clearance CNR, protocol number 0031862/05/04/2018 IFT).

### 4.2. Laboratory Methods

Genomic DNA was extracted from PBMCs using a QIAamp DNA Mini Kit (Qiagen, Hilden, Germany) according to the manufacturer’s instructions. Leukocyte telomere length was measured by monoplex real-time PCR quantitative analysis (monoplex qPCR) on a 7300 real-time PCR instrument (Applied Biosystems, Waltham, MA, USA). This method allows the number of copies of telomeric repeats (T) to be determined, as compared to a single-copy gene (S) used as a quantitative control (T/S ratio) (Cawthon RM, 2002) [38]. Telomere and single-copy gene β-globin (HGB) were analyzed on the same plate to reduce inter-assay variability. DNA (35 ng) was amplified in a total volume of 20 μL containing 10 μL of SYBR Select Master Mix (Applied Biosystems, Waltham, MA, USA); primers for telomeres and the single-copy gene were added to a final concentration of 0.1 μM (Tel Fw), 0.9 μM (Tel Rev), 0.3 μM (HGB Fw), and 0.7 μM (HGB Rev), respectively. Primer sequences were: Tel Fw 5′-CGGTTTGTTTGGGTTTGGGTTTGGGTTTGGGTTTGGGTT-3′; Tel Rev 5′-=GGCTTGCCTTACCCTTACCCTTACCCTTACCCTTACCCT-3′; HGB Fw 5′-GCTTCTGACACAACTGTGTTCACTAGCAAC-3′; and HGB Rev 5′-CACCACCAACTTCATCCACGTTCACCTTGC-3′. The enzyme was activated at 95 °C for 10 min, followed by 40 cycles at 95 °C for 15 s and 60 °C for 1 min. In addition, two standard curves (one for HGB and one for telomere reactions) were prepared for each plate using a reference DNA sample (Control Genomic Human DNA, Applied Biosystems, Waltham, MA, USA), diluted in series (dilution factor of 2) to produce five concentrations of DNA ranging from 50 to 6.25 ng in 20 μL. Measurements were performed in triplicate and are reported as the T/S ratio relative to the calibrator sample to enable comparison across runs. Replicate assays of the same sample were carried out to calculate the interassay variation. The average standard deviation over three different assays was 4.2%. Assuming normal distribution, samples differing in average telomere length by as little as 8.3% (1.96 × SD) should be distinguishable with this method at the 95% confidence interval [38]. No amplification of the negative controls with either primer set (HGB and telomeres) was observed.

### 4.3. Statistical Analysis

Data are presented as mean ± SD unless otherwise indicated. Data from paired groups (e.g., manifest vs. pre-manifest) were compared using the two-sided Wilcoxon signed-rank test, while the unpaired data were compared using the Mann–Whitney test. Correlation was assessed using linear regression and Spearman’s correlation index. Multiple independent groups were compared using the Kruskal–Wallis test and post-hoc analysis. Cut-off points for the receiver operating characteristic (ROC) curves were determined using the Youden index criterion. Statistical analysis was performed using R-Bioconductor (Bioconductor, Boston, MA, USA) and Statistix 8.0 (Analytical Software, Tallahassee, FL, USA). 

## 5. Conclusions

Our previous work [26] suggested LTL as a marker of disease progression in pre-HD patients. This follow-up study was designed to identify in pre-HD patients carrying alleles with different CAG repeat numbers a threshold LTL in a time period close to the disease onset. There was a marked reduction in telomere length in the pre-HD patients about 2.5 years before HD onset compared to the controls and independent of CAG size. This homogeneity allows a common cut-point of 0.70 T/S to be identified between pre-HD and manifest HD. LTL values > 0.70 may indicate a pre-manifest stage at about 3 years before clinical onset, while LTL < 0.70 T/S may indicate imminent clinical onset. Longitudinal studies, e.g., at 5 and 10 years before onset, are warranted to complete the real timing of telomere shortening. 

There are no effective disease-modifying treatments for HD to date, but therapeutical trials investigating therapies to modify the course of the disease have shown promising results. There is, therefore, the need to identify among pre-manifest patients a temporal window more or less close to clinical onset in which to initiate disease-modifying therapies. Overall, our data show that LTL measurement has a robust prognostic value in pre-manifest HD, although it may be of limited relevance for tracking disease progression after clinical onset. Compared to other validated HD biomarkers, LTL measurement, obtained by a non-invasive procedure, may provide an ideal biomarker of HD disease progression [10]. 

## Figures and Tables

**Table 1:** Demographics of controls and HD patients at baseline (T0) and follow-up (T1). Values are expressed as mean ± SD. TMS: total motor score; TFC: total functional capacity; NA: not applicable.

	Controls N = 45	Pre-HD (T0) N = 45	HD (T1) N = 45
Age at blood sampling (years)	41.9 ± 10.5	41.9 ± 10.4	45.3 ± 10.2
Sex (males, %)	32.4	42.1	42.1
Median CAG repeat (range)	NA	43 (39–51)	43 (39–51)
TMS	NA	3.3 ± 2.4	13.2 ± 5.2
TFC	NA	12.5± 1.2	11.8 ± 2.1
Age at onset (years)	NA	NA	44.4 ± 10.7

**Table 2:** Distribution of median LTL (T/S) in HD patients carrying different CAG repeat numbers at baseline (T0) and at follow-up (T1). Values are reported as median (Q1–Q3).

CAG Repeat (n.)	Age at T0 (years)	LTL at T0 pre-HD	LTL at T1 HD	LTL Reduction (T/S)
Total (45)	40 (34–51.5)	0.77 (0.74–0.81)	0.66 (0.63–0.67)	0.12(0.09–0.16)
≤40 (6)	57.5 (55–60.3)	0.77 (0.73–0.79)	0.68 (0.68–0.69)	0.08 (0.07–0.11)
41 (6)	53.5 (46.8–55.3)	0.79 (0.76–0.83)	0.66 (0.64–0.69)	0.13(0.08–0.16)
42 (8)	43.5 (40.3–49.0)	0.80 (0.75–0.86)	0.67 (0.64–0.72)	0.12(0.10–0.14)
43 (7)	36.0 (33.0–44.0)	0.79 (0.75–0.87)	0.65 (0.63–0.66)	0.12 (0.09–0.21)
44 (6)	35.0 (33.8–39.0)	0.74 (0.70–0.77)	0.65 (0.61–0.68)	0.09(0.06–0.12)
45 (6)	32.5 (28.0–37.8)	0.80 (0.74–0.83)	0.61 (0.56–67)	0.18 (0.15–0.21)
≥46(6)	33.5 (28.5–36.8)	0.76 (0.72–0.81)	0.65 (0.63–0.66)	0.13(0.07–0.15)
*p*	*p* < 0.00001	*p* = 0.26	*p* = 0.15	*p* = 0.02

## Data Availability

The data presented in this study are available on request from the corresponding authors. Data are not publicly available due to privacy restrictions.

## Supplementary Materials

The following supporting information can be downloaded at: https://www.mdpi.com/article/10.3390/ijms232113449/s1.
