# Supplementary material for: Leukocyte Telomere Length as Potential Biomarker of HD Progression: A Follow-Up Study
Source: Int J Mol Sci. 2022 Nov 3;23(21):13449. doi: 10.3390/ijms232113449 (PMC9654348; doi:10.3390/ijms232113449)
Supplement: Supplementary file 1 [file ijms-23-13449-s001.zip › ijms-2004944-supplementary.pdf]

Table S1. Correlation (r) between LTL and clinical data at T0 and T1. TMS: Total Motor Score; TFC: Total Functional Capacity)

**Clinical data**	**LTL (T0) (p)**	**LTL (T1) (p)**
TMS T0	-0.10 (0.5)	/
TFC T0	0.08 (0.61)	/
TMS T1	/	-0.09 (0.35)
TFC T1	/	0.12 (0.41)
